# Double shunt technique for hybrid palliation of hypoplastic left heart syndrome: a case report

**DOI:** 10.1186/1749-8090-6-146

**Published:** 2011-10-26

**Authors:** Marcelo Biscegli Jatene, Patrícia M Oliveira, Rafael A Moysés, Ieda Biscegli Jatene, Carlos A Pedra, Simone F Pedra, Fabiana Succi, Vitor Oliveira Carvalho, Carlos R Ferreiro

**Affiliations:** 1 Serviço de cirurgia Cardíaca Pediátrica do Hospital do Coração (HCor), São Paulo - SP - Brazil

**Keywords:** Congenital heart disease, hypoplastic left heart syndrome, Norwood, Infant, Shunts

## Abstract

We report a technique to palliate hypoplastic left heart syndrome, with no PDA stenting, but with double polytetrafluoroethylene shunt from pulmonary artery to ascending and descending aorta by combined thoracotomies. A 30-day-old female was operated with this technique. Five months after first operation, the child was submitted to Norwood/Glenn operation. Good hemodinamic recovery and initial clinical evolution was observed. The child was extubated in 8^th ^post operatory day and reentubated in the next day due to pulmonary infection. Despite antibiotic treatment, the child died after systemic infectious complications.

## Introduction

Different surgical techniques for hybrid procedure for hypoplastic left heart syndrome (HLHS) have being described [1]. Taking into account local fibrosis or tissue friability during second stage operation, the possibility of aortic arch obstruction related to stent deployment and surgical difficulty in management of distal ductal stented area must be considered [2-5].

Caldarone [6] reported an alternative solution to palliate HLHS, with pulmonary artery (PA) to innominate artery shunt, associated to bilateral pulmonary artery banding (PAB), patent ductus arteriosus stenting and atrioseptostomy, not avoiding future events related to the stent.

The purpose of this case report is to describe a new technique to palliate HLHS with banding PA branches, without patent ductus arteriosus stent, but with a double shunt from PA to ascending and descending aorta by combined thoracotomies. This new technique provides adequate flow to aortic arch and descending aorta, avoiding patent ductus arteriosus stenting, and, eventually, future problems with patent ductus arteriosus stent removing.

## Case report

The operation is performed by 2 combined approaches: 1. median sternotomy and 2. left thoracotomy. Cardiopulmonary Bypass is not used. By median sternotomy, right and left PAB with 3.0 mm Polytetrafluoroethylene bands are performed.

**Figure 1:**
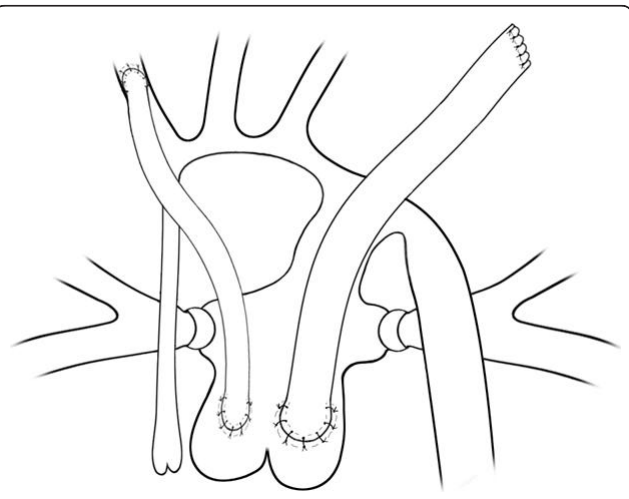
**Double shunts from pulmonary trunk**.

After heparin infusion (2 mg/kg), a reverse Blalock-Taussig shunt with 4 mm Polytetrafluoroethylene graft is performed from proximal PA to inomminate artery. After that, a 7 mm Polytetrafluoroethylene graft is anastomosed in the left side of proximal PA, directed towards left pleura (Figure 1).

**Figure 2:**
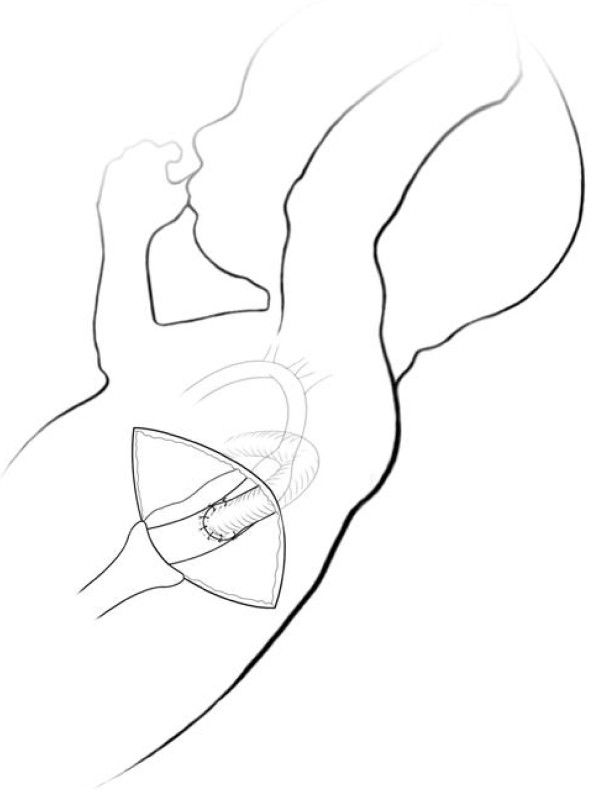
**By left thoracotomy, graft is anastomosed to descending aorta**.

Distal edge of the graft is closed and placed inside left pleura. Chest is drained and closed. The child is then repositioned and left 4^th ^intercostal space is opened; the Polytetrafluoroethylene graft is identified and anastomosed with descending aorta (Figure 2).

A 3.5 kg white female, 30 days old, was admitted; prostaglandin E-1 was initiated, after diagnosis, with 22 days of life. The echocardiogram showed 1.4 mm ascending aorta, small Atrial Septal Defect (3.0 mm), mild tricuspid regurgitation and RV systolic dysfunction.

**Figure 3:**
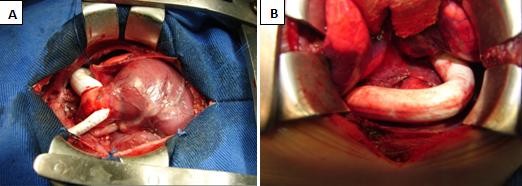
**Illustrations of shunts**. A - Double shunts from pulmonary trunk to innominate artery and towards left pleural space. B - Shunt anastomosed to descending aorta by left thoracotomy.

Bilateral PAB and double shunting procedure with 4.0 and 7.0 mm Polytetrafluoroethylene graft were performed, as described before (Figure 3A and 3B). Oxygen saturation after PAB rised from 82% to 89%. Atrial Septal Defect stenting was required in 1^st ^PO day, due to restrictive Atrial Septal Defect. Warfarin anticoagulation was initiated.

The child experimented a sudden cardiac arrest in 75^th ^PO day. The echo after recover showed important right ventricular dysfunction and 4.0 mm shunt occluded. Retrograde flow to aortic arch from 7.0 mm shunt was demonstrated.

Five months after first operation, a Norwood/Glenn operation was performed; pre-operative mild/moderate right ventricular dysfunction was demonstrated. Cardiopulmonary Bypass was installed, using 2 arterial lines: one with the shunt from PA to descending aorta and the second by a new shunt to innominate artery. Flow was maintained all time during Cardiopulmonary Bypass. Pulmonary artery bands were removed, with no residual stenosis; patent ductus arteriosus was disconnected and reconstruction of aortic arch was performed using a left pulmonary artery homograft, with no ascending aorta reimplantation. Atrial Septal Defect stent was removed and Glenn anastomosis was permormed.

Good hemodynamic recovery was observed (SO_2 _88%), and SVC/PA pressure ranged from 16 to 18 mmHg. Initial evolution was good, with tracheal extubation in 8^th ^post operatory day, being reentubated in the next day. Pulmonary infection was detected and, despite antibiotic treatment, the child died in 12^th ^post operatory day, after systemic infectious complications.

## Comment

Unsuccessful series of hybrid procedures in HLHS previously performed in our institution stimulated us to find a different solution for a surgical proposition that we considered very attractive. We had problems in stent deployment in 1 case, and, in another one, technical problems and tissue tearing with bleeding during patent ductus arteriosus dissection.

Considering the need of ascending and descending aorta flow, we developed the idea to create another shunt from main PA to descending aorta, added to a reverse shunt, as described by Caldarone [6].

We considered the use of two thoracotomies an additional risk of morbidity. In this case, no problems associated with the two approaches were observed, encouraging us to move forward with this idea.

The 4 mm shunt occlusion was really a problem; it's difficult to assume, but the cardiac arrest probably had a relation with the event, causing right ventricular dysfunction and change of directions and timing in the second stage of the treatment. We considered that some competition between the two shunts could be a cause of flow reduction and shunt occlusion. We suggest that the double shunting technique could be more indicated in cases of associated aortic coarctation or severe aortic arch hypoplasia.

Second stage operation was performed, not in the best right ventricular function, but the operation was considered successful. Our initial idea that absence of patent ductus arteriosus stent should facilitate the surgical procedure was confirmed. No problems during patent ductus arteriosus dissection were observed. Cannulation of both shunts as arterial lines, despite the possible presence of debris inside the shunts, must be considered an attractive option.

Unfavorable evolution of the patient was observed, but we consider that infection, and not the technique, was the most responsible for the outcome.

## Consent

Written informed consent was obtained from the patient for publication of this case report and accompanying images. A copy of the written consent is available for review by the Editor-in-Chief of this journal.

## Competing interests

The authors declare that they have no competing interests.

## Authors' contributions

MBJ wrote the draft of the manuscript and obtained the written consent. All authors performed the literature review. MBJ and VOC participated in the manuscript writing. MBJ was the chief surgeon and responsible for finalization of the manuscript. All authors have read and approved the final manuscript.
